# The implications of the feminization of the primary care physician workforce on service supply: a systematic review

**DOI:** 10.1186/1478-4491-12-32

**Published:** 2014-06-04

**Authors:** Lindsay Hedden, Morris L Barer, Karen Cardiff, Kimberlyn M McGrail, Michael R Law, Ivy L Bourgeault

**Affiliations:** 1 Centre for Health Services and Policy Research, University of British Columbia, 201-2206 East Mall, V6T 1Z3 Vancouver, BC, Canada; 2 School of Population and Public Health, University of British Columbia, 2206 East Mall, V6T 1Z3 Vancouver, BC, Canada; 3 Telfer School of Management and Institute of Population Health, University of Ottawa, 1 Stewart St, K1N 6 N5 Ottawa, ON, Canada

**Keywords:** Primary health care, Physicians, Gender differences, Health human resources planning, Workforce planning, Practice patterns, Female, Male

## Abstract

There is a widespread perception that the increasing proportion of female physicians in most developed countries is contributing to a primary care service shortage because females work less and provide less patient care compared with their male counterparts. There has, however, been no comprehensive investigation of the effects of primary care physician (PCP) workforce feminization on service supply. We undertook a systematic review to examine the current evidence that quantifies the effect of feminization on time spent working, intensity and scope of work, and practice characteristics. We searched Medline, Embase, and Web of Science from 1991 to 2013 using variations of the terms ‘primary care’, ‘women’, ‘manpower’, and ‘supply and distribution’; screened the abstracts of all articles; and entered those meeting our inclusion criteria into a data abstraction tool. Original research comparing male to female PCPs on measures of years of practice, time spent working, intensity of work, scope of work, or practice characteristics was included. We screened 1,271 unique abstracts and selected 74 studies for full-text review. Of these, 34 met the inclusion criteria. Years of practice, hours of work, intensity of work, scope of work, and practice characteristics featured in 12%, 53%, 42%, 50%, and 21% of studies respectively. Female PCPs self-report fewer hours of work than male PCPs, have fewer patient encounters, and deliver fewer services, but spend longer with their patients during a contact and deal with more separate presenting problems in one visit. They write fewer prescriptions but refer to diagnostic services and specialist physicians more often. The studies included in this review suggest that the feminization of the workforce is likely to have a small negative impact on the availability of primary health care services, and that the drivers of observed differences between male and female PCPs are complex and nuanced. The true scale of the impact of these findings on future effective physician supply is difficult to determine with currently available evidence, given that few studies looked at trends over time, and results from those that did are inconsistent. Additional research examining gender differences in practice patterns and scope of work is warranted.

## Background

The primary care physician (PCP) workforce in many industrialized nations is increasingly female. In several industrialized countries, the proportion of PCPs who are women has doubled or nearly doubled over the last 30 years [1,2]. Globally, 32% of all physician graduates worldwide are female, and that percentage is higher, on average, in family medicine [3]. Thirty-four percent of family medicine/general practice physicians and 55% of family medicine residents in the United States (US) are women [4]. In Canada, women now make up 58% of medical school enrollees (up from 14% in 1968) [5] and more women than men are choosing to specialize in primary care [6].

Amidst often highly-charged claims of physician shortages from the public and medical leadership alike, future physician workforce planning has been identified as a priority for both research and policy action in many industrialized countries, and is essential for the rational management of health care systems [7]. If they are to be an effective policy tool, physician workforce planning will need to go beyond simply projecting the traditional factors of population growth and ageing, and physician headcounts [8-10], to include variables that affect both service requirements (population need) and availability [11-13].

The rapid feminization of the PCP workforce over the past half-century is a significant demographic change that has the potential to influence service availability. For example, claims that changes in the gender balance of the PCP workforce will change the effective overall supply of primary health services (for example, because female physicians work fewer hours than their male counterparts) and/or the mix of available services (for example, because of differences in styles of practice) have a certain intuitive validity. Unfortunately, debate in this arena has, for the most part, not advanced much beyond these simplistic claims.

Thus far, even where workforce planning models account for changes in physician workforce demographics (such as feminization), they commonly apply a simplistic calculus, using simple service or headcounts, or assuming the work of a female physician as a fixed proportion of a male physician (typically using full-time equivalent measures) [14,15]. It is very difficult to find supply projection models that embody evidence about the differences between male and female physicians in life-course productivity, changes over time in trends in retirement, or recent changes reflecting shifting work-life priorities amongst younger cohorts of physicians. The focus of this paper is to synthesize the evidence relating to the first of these factors - male-female differences in physician service provision over a life-cycle. Our specific population of interest is general practice and family medicine (which we will henceforth refer to as PCPs); other primary care specialties such as internal medicine and pediatrics will be discussed in a subsequent manuscript.

This systematic review examines evidence related to the effect of the PCP workforce, defined here as feminization on the supply of physician services. Specifically, we reviewed studies that compared male and female PCPs in terms of the amount of time they spent working, how intensely they worked (that is the number of services or patient encounters per unit time), and whether their practice and service characteristics differed.

## Methods

### Search strategy and inclusion criteria

In an effort to ensure comprehensiveness, we used multiple search strategies to locate both peer-reviewed and grey literature sources. Peer reviewed literature was selected from Medline (OVID), Embase, and Web of Science. We limited our search to English language articles published between January 1990 and January 2013. Our database-specific search terms included variations on ‘physician’, ‘women’, and ‘workforce’ (see Additional file 1 for the full search strategies). We identified relevant grey literature using the Canadian Health Research Library, ProQuest Dissertations and Theses, and the Canadian Health Human Resource Network Library (http://www.hhr-rhs.ca/index.php?option=com_content&view=article&id=168&Itemid=78&lang=en). We also conducted searches of the websites of organizations, groups, governments, associations, and professional bodies identified using the Canadian Agency for Drugs and Technologies in Health’s ‘Grey Matters’ guide to grey literature [16]. Additionally, we completed forward and reverse citation searches (snowballing) of included peer-reviewed articles using Google Scholar.

We imported search results into a reference manager and removed any duplicates. We screened all abstracts for relevance to the research topic and pulled relevant articles. Two reviewers independently reviewed all full-text articles using the inclusion and exclusion criteria in Table 1 and thematic typology in Table 2, and disagreements were resolved by discussion. We computed a Kappa statistic for inter-rater reliability. Studies were not excluded due to quality issues; however, methodological concerns are presented as part of both the Results and Discussion sections.

**Table 1:** Inclusion and exclusion criteria

**Inclusion criteria**	**Exclusion criteria**
Publication Details	
Published between January 1990 and January 2013; published in English	Published before January 1990 or after January 2013; published in a language other than English
Participants/Population	
PCPs (studies focusing on all physicians were included only if results pertaining to PCPs were presented separately)	Other physician specialties; all physicians, where separate analysis for PCPs is not presented
Comparison	
Male to female PCPs^1^	Does not compare male and female physicians
Outcome Measures	
A measure of one or more of the following: time spent working, intensity of work, scope of work, or practice characteristics^2^	None of time spent working, intensity of work, scope of work, or practice characteristics
Design	
Original research	Editorials, comments or commentaries, letters; reviews articles; reports with no primary data analysis

^1^Specialist physicians (such as pediatricians, or general internists) who may practice like PCPs on occasion (that is acting as a point of entry to the health care system, providing person-focused care over time, and acting as a coordinator for care provided elsewhere) were not included.

^2^Raw or adjusted results for one or more of these measures must be presented. If these measures were included as covariates in a multivariate modeling exercise (for example, for income), the study was excluded unless raw comparisons on one of these outcomes are also presented.

PCP, primary care physician.

**Table 2:** Article typology

**Theme**	**Subtheme**	**Potential effect on supply - Direct/Indirect**
Years of practice	• Retirement	Direct - for example, shortening of career or more lengthy absences from practice
	• Leaves of absence	
Hours of work	• Full- versus part-time work	Direct **-** for example, less time spent working overall, or less time spent on direct patient care in favour of other responsibilities
	• Time spent on patient care	
	• Time spent on administrative responsibilities, professional development	
Intensity of work	• Number of services/time	Direct - (lower service or patient volumes)
	• Number of patients/time	
Scope of work	• Patient characteristics	Indirect - (restrictions in scope of practice, or basket of services delivered; restricted patient population; reduced availability of out-of-office or off-hours care)
	• Service provision	
Practice characteristics	• Location	Indirect - (imbalance between urban- versus rural-based practices leading to shortages in some areas, oversupply in others)
	• Group practice versus solo practice	

### Data extraction and article typology

We abstracted and summarized the following data from all included articles: citation; country; objectives; study sample, response and drop-out rates (where applicable); study design (cross-sectional or longitudinal); data collection (administrative, survey, or other primary data); analytic methodology; outcome measure(s); and results.

We coded articles using a typology designed with the intention of capturing any practice differences between male and female physicians that could, either directly or indirectly, affect the availability of primary health care services. It includes variations in what care is delivered, to whom, and how much. The typology consists of five themes and eleven subthemes (Table 2). Table 2 includes examples of how each thematic area may be linked to changes in service availability.

We conducted a qualitative examination of study quality by assessing the following items: clarity of research questions and objectives; appropriateness of study design; sample size and representativeness; validity of measures; addressing possible confounders; and generalizability.

## Results

### Search results

**Figure 1:**
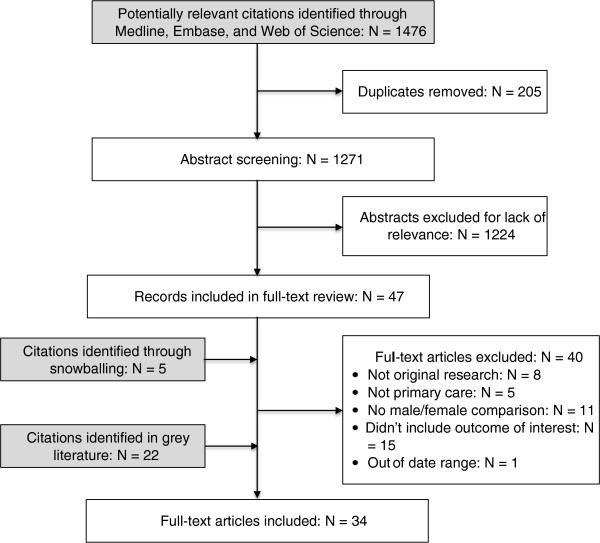
Search results.

The initial search of Medline, Embase and Web of Science located 1,476 citations, of which 205 were duplicates. The abstracts from the remaining 1,271 were screened for relevance to the topic, and 1,224 were excluded, leaving 47 peer-reviewed articles. An additional 27 studies were identified from grey sources and through snowballing of references in selected articles. These 74 sources were retained for full-text review (Figure 1). Of these, 34 studies met the inclusion criteria; they are summarized in Additional file 2. The K-coefficient for inter-rater agreement beyond change was 0.84.

Thirty of the 34 included studies (88%) had been published in peer- reviewed fora. Fifteen of the 34 (44%) were conducted in Canada, four (12%) in the US, and five (15%) in the United Kingdom. Twenty-seven studies (79%) used a cross-sectional methodology. Of these, 21 (78%) used retrospective survey data, five (19%) used administrative data, and one employed prospective primary data collection. Of the seven (21%) studies that used longitudinal methods, four (57%) used administrative data, one combined administrative and survey data, and two (29%) used surveys alone.

### Thematic results

**Figure 2:**
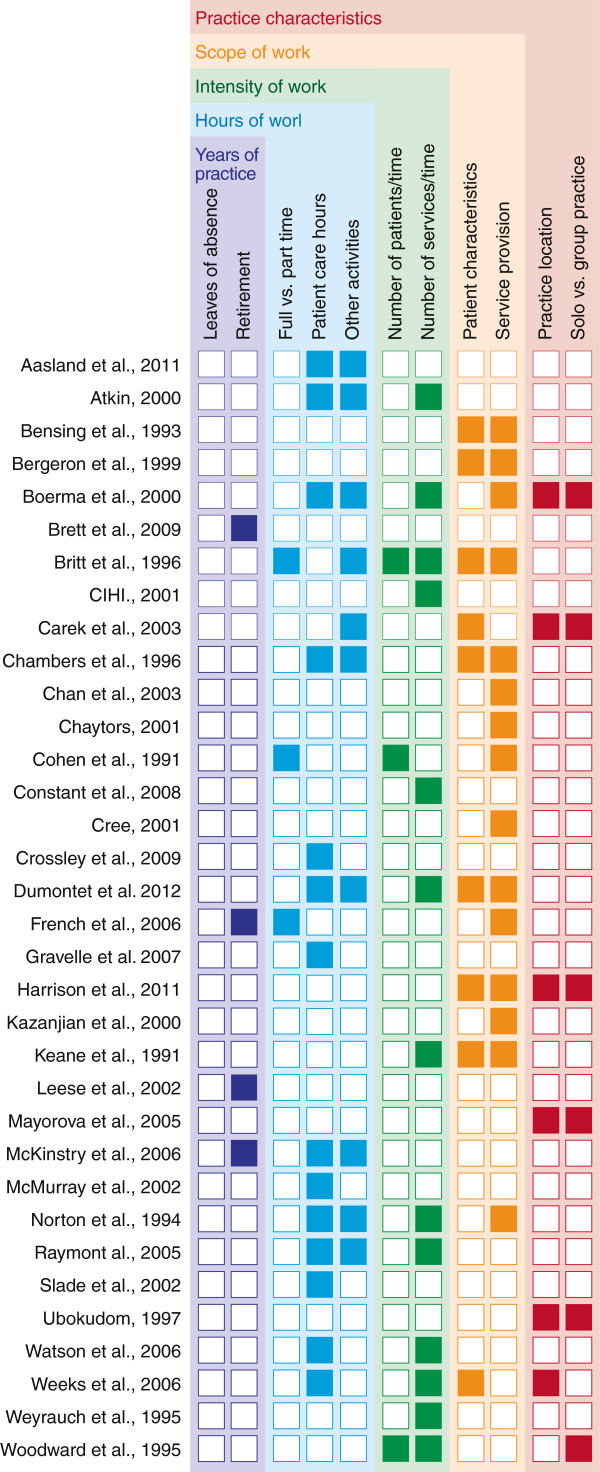
Frequency of thematic categories.

Hours of work, intensity of work (defined here as number of services or patient encounters per unit time), and scope of work featured in 18 (53%), 14 (42%) and, 17 (50%) studies respectively (Figure 2). Practice characteristics were examined in seven (21%) studies, and years of practice was a focus in only four (12%). Themes with a direct impact on service availability (years of practice, hours and intensity of work) were more commonly featured (26 articles, 76%) than those that affect supply or availability of services indirectly (practice characteristics, scope of practice) (18 articles, 53%). Slightly more than sixty percent of the included studies focused on a single thematic area.

#### ***Hours of work***

All 18 studies that examined hours of work found that female PCPs tended to self-report working fewer hours than their male counterparts. Few of these studies, however, presented results that adjusted for physician age, practice characteristics or other factors that may confound the relationship between physician sex and work hours (for example, [17,18]). In their survey of English general practitioners, Gravelle and Hole found that the average difference in hours per week worked between males and females was 11.8 hours [17]. Forty-five percent (5.3 hours) of this difference was due to the greater proportion of male PCPs at each age working full-time, and 46% (5.4 hours) was due to female PCPs reducing their hours more than male PCPs who have the same family circumstances. The final 9% (1.1 hours) of the difference was due to differences in physician demographics (for example, age) and practice characteristics (for example, size of practice) [17].

In their European study, Boerma and van den Brink-Muinen found that, on average, male PCPs worked more hours per week, excluding on-call time (45.1 versus 36.2) [18]. In countries where the difference in hours was statistically significant (12 of 32 study countries), male PCPs worked more in ten, and female PCPs worked more in two [18]. Results from North America are similar, with female PCPs working between four and 14.5 fewer patient-care hours per week [8,19-23].

Female PCPs were more likely to report working part-time (31.6% versus 11.1%) [17,24], and billed Canadian provincial health insurance plans for fewer months of the year [25]. Having children under the age of 18 increased the probability that female PCPs worked part-time, but had no effect on male PCPs [17].

Despite consistent differences found in hours worked overall, and specifically in hours spent on patient care, male and female PCPs tended to spend a similar amount of time on-call [21,23,26].

Three of the included studies examined longitudinal trends in work hours for male and female physicians [8,27,28]. In their study on PCP labour supply in Canada, Crossley *et al*. found a secular decline in hours of patient care between 1982 and 2003 [28]. Although female physicians were found to have worked fewer hours than male physicians, a change in the behavior of male PCPs accounted for a greater proportion of the decline in hours of patient care than did the growing proportion of females in the workforce. The gap in hours worked between male and female PCPs diminished over the study period [28]. They also reported that, for female physicians only, there was a significant age effect on hours of patient care: hours declined up to approximately age 38, and then gradually increased with age [28]. This would be consistent with a ‘childbearing years’ effect. Aasland and Rosta found that the gap between male and female PCPs’ hours of work is also narrowing in Norway, with female PCPs having worked significantly fewer hours than male PCPs between 2000 and 2006, but not in 2008 [27]. In that country, however, physicians’ hours have, on the whole, increased rather than declined, with the increase in hours obviously being more marked amongst female physicians [27].

#### ***Intensity of work***

Eleven studies compared the number of services per unit of time delivered or number of patients seen for male and female PCPs. Of these, five presented multivariate results, controlling for the effect of physician and patient characteristics, or other confounders.

Cohen *et al*., Woodward and Hurley, and the Canadian Institute for Health information all found that Canadian male PCPs bill for more services compared with their female colleagues, and that physician gender contributed significantly to explaining variation in service activity [25,29,30]. Boerma and van den Brink-Muinen similarly found that European female PCPs have on average 4.1 (or 14%) fewer office contacts per day. This difference in office contacts was only significant in 12 of the 32 study countries, and in half of these, female physicians had significantly more daily contacts than male physicians [18]. Additionally, when results were restricted to only include physicians who worked full-time, the sex-related difference in contacts dropped to 2.3 fewer contacts per day for female physicians, and a significant difference was found in only six of 32 countries. Of these, women had significantly more contacts per day in three [18].

Consistent with the age-stratified results repented for hours worked, Constant and Legere reported that the difference between male and female PCPs peaks between the ages of 36 and 40, and declines thereafter [14].

Unadjusted results from the remaining studies were relatively consistent: male PCPs were reported to deliver more services than female PCPs (700 versus 399/month) [24], and to have more patient encounters (between 32 and 72/week) (for example: [19,21,26]). Female PCPs, however, were found to manage more problems per patient encounter (157.8 versus 145.4 per 100 encounters) and spend 40% more time with each patient (20.5 versus 14.4 minutes) [31,32].

In their longitudinal examination of intergenerational differences in workloads of physicians from six Canadian provinces, Watson *et al*. found that between 1992 and 2001, female PCPs reduced their workloads (defined as number of visits per year) by 6.1%, while male workloads remained stable. The result was an accentuated difference in workload over time: female physicians’ workloads were, on average, 74% of the workloads of their male counterparts in 1992, and 68% in 2001 [8].

These results run somewhat counter to those reported by Crossley *et al*. who found that the gap in self-reported hours worked between male and female physicians was narrowing [28]. It is possible that these conflicting results could be caused by some combination of differences in time periods used for analysis (1982 to 2003 versus 1992 to 2001), outcome measure (hours versus billed consultations) or other differences in methodology [28]. If one takes both sets of results at face value and attempts to reconcile them, a possible conclusion would be that male PCPs are reducing their hours while maintaining visit counts, while female PCPs are maintaining their hours, but are decreasing their visits. Taking account of other results cited here, it may be that female PCPs are simply changing their style of practice, taking more time with each patient and dealing with more problems per visit. The other conclusion that can be drawn from these results is that measuring physician productivity is difficult, and that the numerator (outputs or outcomes per unit of activity) matters [33].

#### ***Scope of work***

##### 

**Patient characteristics** Compared with male PCPs, female PCPs saw a higher proportion of female patients [24,25,31,34] in all age groups [27], but especially in the 15 to 49 age category [24,25]. They also saw fewer older-aged patients than their male counterparts [1,23]. These results survived multivariate analyses that accounted for the age of physician, practice location, and graduation period [25].

##### 

**Care delivered** Controlling for patient and physician demographics, female PCPs were significantly more likely to manage issues related to the reproductive or female genital system [1,31,34], as well as psychological and social problems [1,31,34]. Female physicians were less likely to manage issues of the musculoskeletal, or male genitourinary systems [1,31].

With respect to obstetrical and prenatal care, results from US-based literature were inconsistent with those from Canada. In the US, male and female PCPs were equally likely to provide prenatal care, with or without delivery [23]. In contrast, in Canada, female physicians were more likely than their male counterparts to provide prenatal care, but were less likely to provide intrapartum care [24].

After adjusting for problems per encounter, as well as physician, practice and patient characteristics, Australian male PCPs had a higher rate of prescribing (4.3% more medications per 100 patients) [1]. Female PCPs recorded 19.5% more clinical treatments (for example, education and counselling), 18.5% more referrals, 8.1% more imaging ordered and 9.6% more pathology tests ordered [1]. In their 1993 study on service delivery trends for male and female PCPs in the Netherlands, Bensing and colleagues found that female physicians wrote fewer prescriptions and performed fewer technical interventions compared with male physicians; however, they ordered more laboratory tests [34]. They found no difference in the rate of referrals to specialists [34].

Chan and colleagues examined the referral rates for Canadian male and female PCPs. Like Harrison *et al*. [1] they found that female physicians referred to specialists about 10% more frequently than their male colleagues after making adjustments for patient age and gender [35].

Boerma and van den Brink-Muinen found that male European PCPs were more involved in technical procedures; however the difference was smaller in countries with a gatekeeping system [18].

##### 

**Out-of-office and Off-hours care** Five studies examined the provision of out-of-office and/or off-hours care [18,23,24,26,36]. In 1991, Keane *et al*. reported that a smaller proportion of Canadian female than male PCPs billed for home visits (1.5 versus 3.7 per 100 patients) and after hours care (7.0 versus 9.6 per 100 patients, after controlling for the effects of place and date of MD graduation, practice location, certification status, and work status [24].

Adjusted for patient, physician and practice characteristics, male PCPs also more routinely made long-term care facility visits (50.6% versus 35.5% for females), and home visits (49.0% versus 33.8% for females) [18]. Male PCPs were also more likely than their female counterparts to bill for time in the hospital (14.8% versus 13.1%, emergency room (37.0% versus 14.2%), or for surgical assists (64.8% versus 47.2%) [18].

Consistent with the multivariate results from Keane *et al*. and Boerma and van den Brink-Muinen, the two studies that report only bivariate results found that female PCPs were less likely to provide after-hours services [23,26], make house calls (for example, 12.7% versus 15.2% for men), and spend significantly more of their work time in office or clinic practice (87.9% versus 80.9% for men [23]. This is in contrast to findings reported by Bergeron *et al*. who report that although male physicians make more home visits compared with female physicians, they spend an almost equal amount of time on this activity (5.7 versus 5.2 hours/week) [36].

#### ***Years of practice***

Patterns of retirement (or practice leave) were examined in four of the included studies [37-40], and results are mixed. French *et al*. found that a similar proportion of male and female PCPs in Scotland intend to retire at age 59 [40]. In their study of Australian physicians, Brett *et al*. report that male PCPs were more likely to intend to retire before age 65: 75% of women compared with 59% of men reported that they intended to work to normal retirement age (rather than retiring early) [37]. In their survey of physicians who had recently left practice, however, Leese *et al*. found that female leavers tended to be younger, and to have children under the age of 18 [38]. This suggests that childrearing responsibilities play a key role in decisions to leave practice, and that female PCPs are more likely to leave practice for reasons other than full retirement, compared with their male counterparts.

Leaves of absence, for reasons of childbearing or otherwise, were not a focus in any of the articles included in this review.

#### ***Practice characteristics***

Female PCPs practicing across Europe and in Australia were less likely than men to work in solo practice (rather than in small or large groups (Europe: 27% of women found to work in solo practice versus 45.2% of men [18]; Australia: 4.6% of women work in solo practice, versus 13.2% of men)) [1]. In the US, male and female PCPs are about equally likely to practice within a small group (32.7% versus 38.3%) [23].

Female PCPs practicing in Europe were significantly less likely to practice in rural areas compared with their male counterparts (14.9% versus 27.2% rural). In contrast, in the US and Australia, women and men were equally likely to choose rural practice [1,23]. Female PCPs in Europe were more likely to work in inner city locations (33.7% versus 18.0%) [18].

## Discussion

The intent of this systematic review was to examine the impact of the increasing proportion of women in the PCP workforce on service delivery in five areas that could affect such projections of service supply: years of practice, hours of work, intensity of work, scope of work, and practice characteristics. Compared with their male colleagues, female PCPs:

• Self-report fewer hours of work (excluding on-call time)

• Have fewer patient encounters, and deliver fewer services (perhaps as an artifact of working fewer hours), but spend longer with their patients during a contact and deal with more separate presenting problems during each visit

• Write fewer prescriptions, but order more laboratory tests, and refer patients on to specialists more frequently

• See more female patients and fewer geriatric patients

• Provide less out-of-office (including home, nursing home and hospital visits) and off-hours care

The scale of the impact of these findings on future effective physician supply is difficult to determine with currently available evidence, given that very few studies looked at time trends or years of practice, and results from those that did are inconsistent. Also, the full impact will depend critically on future trends in the feminization of the workforce. In Canada, and in the UK and other parts of Europe, the proportion of medical students who are female ensures that the overall supply of physicians will continue to become increasingly female in the near term.

Given that fact, the differences in practice patterns between male and female PCPs could result in increased derived demand for specialist physician services, laboratory technicians, imaging technicians or other health professionals, outside of primary health care. The fact that female PCPs spend less time in off-hours care, and are less likely to serve patients at home and in nursing homes, could increase the reliance on already-stretched emergency departments and walk-in clinics as a source of primary health care, and force a rethinking of how medical care is delivered to patients outside standard office hours and locations.

It is important to consider the effects of childbearing and childrearing, which were mentioned in several studies, but were seldom explicitly investigated, and were not the primary focus of any of the research documents reviewed here. Female PCPs who had children under age 18 worked fewer hours per week and were more likely to have self-reported part-time status compared with women who did not. The dampening effect of children on work hours was twice as large for women as it was for men. And, one study found that once family circumstances were accounted for, the gender of the physician had no significant effect on hours worked [17].

An important issue that was not covered in any of the literature reviewed here is the balance between work and household responsibilities among physicians. One study found that female physicians spent more time on unwaged childcare and household jobs than male physicians [41]. Once unwaged household responsibilities were accounted for, female PCPs who have children worked an average of 90.5 hours a week, compared with 68.6 hours per week for males with children [41].

### Consistency of results

Results were strongly consistent across some of the thematic areas, and relatively less so in others. In particular, results relating to the hours and intensity of work were consistent across studies. In other areas, such as practice characteristics, results were highly variable.

The results of this review demonstrate that the drivers of observed differences between male and female PCPs are complex and nuanced. The size of an observed gender difference varied based on the characteristics of the health care system under study and on whether the possible confounding effects of physician age, practice characteristics, and in particular, family characteristics and part-time status were adequately controlled. There were at least 36 different health care systems represented by the studies included in this review. Inconsistent results across studies may be caused by health care system differences including, but not limited to, physician remuneration mechanisms and policies, the gatekeeping role of general practitioners, and general employment policies. An exploration of the role of such system differences was well beyond the scope of this review, but is an important area for future research.

Inconsistent results could also be a function of methodological and measurement differences across studies, and whether the confounding effects of other physician, patient, and practice characteristics have been accounted for. For example, gender differences in the number of patient contacts per day disappeared once full- versus part-time status had been accounted for in work by Boerma and van den Brink-Muinen [18]. Differences in hours worked depended on whether auxiliary activities such as on-call time were included as part of ‘hour worked’ [23]. Similarly, differences in care provision were attenuated once patient characteristics and practice location was accounted for (for example, [1,31]).

### Methodological issues

As part of our qualitative assessment of study quality, we identified some significant methodological concerns with the studies included in this review. For the most part, they relied on cross-sectional retrospective surveys. Such surveys are always subject to recall bias, though unless there were systematic male versus female differences in accuracy of recall, this may not be an issue in this particular circumstance. But surveys do tend to produce inflated estimates of hours worked for those who report high hours (more often male physicians) and deflated estimates for those reporting low hours (more often female physicians), which may exaggerate any true gender difference [42]. Many studies relied on small, often unbalanced samples, raising concerns about selection bias. All but one study failed to adjust statistically for multiple comparisons, despite conducting as many as 155 separate statistical significance tests [32].

Perhaps even more concerning, however, is that 12 (35%) studies presented only unadjusted, bivariate results, failing to control for the potential confounding effects of other physician, patient or practice characteristics (for example, [23,26,34]). Additionally 6 (18%) undertook only rudimentary stratification (for patient age and gender, for example) (for example, [24,25,34,43]). Statistical methods controlling for confounders may not yet have been accepted practice in this field when some of these earlier papers were published, which may explain their limited use. Comparisons between adjusted and unadjusted results suggest that physician age, family characteristics and practice location, at a minimum, can have important influences on apparent male-female differences in key practice and productivity indicators. For example, older physicians - who are more likely to be male - tend to see more older patients [18], and physicians who work in rural-based clinics practice differently from physicians who practice in urban centres [32]. Thus the impacts of physician age and practice location may be conflated with a gender effect in unadjusted analyses, since female PCPs tend to be younger [31] and more likely to work in urban centres in some countries [18].

### Gaps in knowledge and future research

Given the reliance on cross-sectional and survey data, and the relative underutilization of longitudinal or administrative datasets in this area, there remains a need to critically examine activity levels, over time and at a population level, adjusting for the potentially confounding effects of age and cohort. The issue of retirement patterns has also not been adequately examined with reference to the effects on time spent working. It is possible, for example, that although female PCPs work less, especially around childbearing years, they may retire later than their male counterparts, reducing or even eliminating a career difference in time spent working. While historically this may not have been true, trends over time suggest that it might become so in future. The key point is that differences in retirement patterns between male and female physicians may partially or wholly offset other trends in service provision, when viewed over an entire life-cycle. Leaves of absence taken for parental or other reasons should also be examined for their effects on both time and intensity of working. No studies included in this review examined absences from practice.

To date, the literature examining other practice differences between male and female physicians that could have an important impact on health human resources planning has been limited. More studies comparing the patient populations of male and female PCPs - beyond simple gender concordance and patient age - are certainly warranted. Specifically, very little work has been done examining differences in patient morbidity levels, or chronic disease burdens. Additionally, more nuanced investigations of service mix, problems seen, and care delivered would address currently unanswered, but important, questions bearing on the future provision of physician services. For example, differences in practice style between male and female physicians have currently received little attention beyond comparisons of time taken for each appointment.

Issues of work-life balance and childrearing and household responsibilities are also under-researched, especially given their observed impact on full- versus part-time job status and working hours [17,41]. In the 2007 and 2010 Canadian National Physician Surveys, the majority of respondents identified attaining balance between personal and professional life as the most important factor for a satisfying practice [44]. Physicians, regardless of gender, are increasingly (and not unreasonably) seeking a work environment that provides this balance, without compromising the quality of care they provide to their patients [45]. Secular trends in time made available for clinical practice obviously have direct implications for projections of physician service provision.

### Limitations

This systematic review used comprehensive search strategies encompassing multiple peer-reviewed and grey literature sources to maximize capture of relevant articles and minimize publication bias. The restriction of articles to those published in English and within the last 23 years may have eliminated some potentially relevant studies. Additionally, because the area of research is not yet well-indexed and the specific topic area is broad, some studies that would be relevant, but whose main comparison was not male versus female PCPs, may have been missed.

Our decision to include only those studies that focused on PCPs, defined here as general practitioners or family medicine specialists, (rather than also including other specialists like general internists or pediatricians - who may practice like PCPs under certain circumstances) may limit the generalizability of our results, particularly with respect to research from the US.

An additional limitation is the decision not to eliminate studies that were deemed of poor quality. The methodologies employed in many of the studies is certainly far from ideal, with many relying on small, unbalanced samples, retrospective surveys, and incomplete (or no) control for the impact of confounding factors. These studies were, however, retained in the review since none of the 30 included would have achieved the level of guidance required for formal guidelines (for example, those issued by the Cochrane Collaboration) and, thus, there was no straightforward way to gauge methodological quality.

Meta-analytic techniques could have been a useful way to summarize the research within individual thematic and subthematic areas; however, small numbers and the variance in outcome measures even within individual subthemes were too great to allow for the use of those tools.

### Implications for health human resource planners

Projections of physician supply must take into account variables other than estimated future physician headcounts. At a minimum, more robust measures that account for gender differences in service volumes, but that also address the implications of the differences in patient mix, service mix, and practice style between male and female physicians need to be developed and used as evidence in these areas becomes available. Other demographic and workforce factors, such as the impact of physician age and cohort - should also be considered.

## Conclusions

Compared with their male counterparts, female PCPs spend less time working, and deliver less care. Evidence as to whether this gap is narrowing is mixed. The effect of childrearing is critically important, affecting female PCPs far more than their male counterparts, in terms of impact on participation in clinical practice. Once the effect of family characteristics has been accounted for, sex has no effect on time spent working. Issues of work-life balance, caregiving and childrearing responsibilities warrant attention in future research.

The literature focuses heavily on differences in the amount of work done by female compared with male physicians, and is almost exclusively based on retrospective surveys with some significant methodological limitations. These studies tell us nothing about differences in the appropriateness or quality of care. Also, more research examining differences in practice characteristics, and patient/service mix, is warranted in order to support the development of robust forecasts of physician supply. Such forecasts would ideally take into account sex-related differences in volume, bct also the implications of the differences in patient/service mix and practice style, and temporal trends in each of these. The extant literature suggests that secular trends in hours of work may dominate sex-related differences in service provision.

## Abbreviations

PCP: primary care physician; US: United States.

## Competing interests

The authors declare that they have no competing interests.

## Authors’ contributions

All authors contributed to the collection and interpretation of the data. LH developed the search and abstraction tools. LH and KC selected studies for review and conducted the abstraction and interpretation of the data. MB, IB, KM, and ML contributed significantly to the analysis and interpretation. LH wrote the first draft and managed subsequent drafts with revisions from all other authors. All authors give final approval of the publication of this version of the paper.

## Supplementary Material

Additional file 1 Medline Search Strategy. Click here for file

Additional file 2 **Summary of Included Studies [**[1]**,**[2]**,**[8]**,**[14]**,**[17]**-**[32]**,**[34]**-**[40]**,**[43]**,**[46]**-**[51]**].** Click here for file
