# Supplementary material for: The implications of the feminization of the primary care physician workforce on service supply: a systematic review
Source: Hum Resour Health. 2014 Jun 4;12:32. doi: 10.1186/1478-4491-12-32 (PMC4057816; doi:10.1186/1478-4491-12-32)
Supplement: Additional file 1 — Medline Search Strategy. [file 1478-4491-12-32-S1.docx]

**Additional file 1**

1. **Medline Search Strategy**

Database: Ovid MEDLINE(R) In-Process & Other Non-Indexed Citations and Ovid MEDLINE(R) <1946 to Present>

Search Strategy:

--------------------------------------------------------------------------------

1 exp Physicians, Family/ (14492)

2 Physicians, family.tw. (181)

3 exp Physicians, Primary Care/ (796)

4 physician, primary care.tw. (25)

5 exp General Practitioners/ (1277)

6 General practitioner.tw. (13510)

7 primary care provider.tw. (1322)

8 primary care practitioner.tw. (222)

9 1 or 2 or 3 or 4 or 5 or 6 or 7 or 8 (30311)

10 exp Women/ (28912)

11 (wom#n or female$).tw. (1229563)

12 10 or 11 (1240645)

13 9 and 12 (3525)

14 exp Health Manpower/ (10931)

15 exp Workload/ (14298)

16 exp Physician's Practice Patterns/ (37643)

17 income/ or exp remuneration/ or exp "salaries and fringe benefits"/ or exp family leave/ or exp sick leave/ (45296)

18 ("labor supply" or "labour supply").tw. (334)

19 "workforce planning".tw. (405)

20 workforce.tw. (10410)

21 "health manpower".tw. (774)

22 workload.tw. (13862)

23 "physician* practice patterns".tw. (254)

24 ("income" or "remuneration").tw. (54333)

25 14 or 15 or 16 or 17 or 18 or 19 or 20 or 21 or 22 or 23 or 24 (168916)

26 13 and 25 (632)

27 limit 26 to (english language and yr="1990 -Current") (537)

***************************

1. **Embase Search Strategy**

Database: Embase <1974 to 2013 April 04>

Search Strategy:

--------------------------------------------------------------------------------

1 exp general practitioner/ (55906)

2 general practitioner.tw. (17578)

3 family physician.tw. (4339)

4 primary care physician.tw. (5104)

5 primary care practitioner.tw. (262)

6 primary care provider.tw. (1702)

7 female/ (5758352)

8 female$.tw. (752338)

9 wom#n.tw. (930494)

10 1 or 2 or 3 or 4 or 5 or 6 (74482)

11 7 or 8 or 9 (6060729)

12 exp health care manpower/ (9811)

13 exp workload/ (25660)

14 exp income/ or exp physician income/ or exp remuneration/ or exp salary/ or exp "salary and fringe benefit"/ (56441)

15 ("labor supply" or "labour supply").tw. (284)

16 "workforce planning".tw. (447)

17 workforce.tw. (11719)

18 "health manpower".tw. (1057)

19 workload.tw. (17748)

20 "physician* practice patterns".tw. (292)

21 12 or 13 or 14 or 15 or 16 or 17 or 18 or 19 or 20 (109823)

22 10 and 11 (22125)

23 21 and 22 (915)

24 limit 23 to (english language and yr="1990 -Current") (820)

***************************

1. **Web of Science**

((TS = (("primary care") OR ("family practice") OR ("family physician*") OR ("primary health care") OR ("general practice")) AND TS = (("sex differences") OR ("gender differences"))) AND Language=(English) AND Document Types=(Article)

*Databases=SCI-EXPANDED, SSCI, A&HCI Timespan=1980-01-01 - 2013-01-17*

*Lemmatization=On*
