# Supplementary material for: The implications of the feminization of the primary care physician workforce on service supply: a systematic review
Source: Hum Resour Health. 2014 Jun 4;12:32. doi: 10.1186/1478-4491-12-32 (PMC4057816; doi:10.1186/1478-4491-12-32)
Supplement: Additional file 2 — Summary of Included Studies [[1],[2],[8],[14],[17]-[32],[34]-[40],[43],[46]-[51]]. [file 1478-4491-12-32-S2.docx]

**Additional file 2:** Summary of Included Studies

| **First Author** (year) | Objective | Sample (Response Rate), **Country** | Design (Data) | Relevant Outcomes | Main Findings/Conclusions | Ref |
| --- | --- | --- | --- | --- | --- | --- |
| **Aasland** (2011) | To describe weekly working hours of Norwegian GPs between 2000 and 2008 as well as the length of patient lists and perceived workload | 227-316 for each time point (19-27%),\|  **Norway** | Longitudinal (Survey) | - Hours per week with patients Hours per week in meetings - Hours per week doing paperwork - Hours per week updating professional skills | - Women work few hours per week than men for the 2000, 2004, 2006 but not 2008 surveys - The average weekly working hours of GPs increased from 2000 to 2008, but only among women GPs | [27] |
| **Atkin** (2000) | To examine the gendered income for family physicians in Ontario, using a mailed survey in 1996. | 262 Ontario PCPs (56.7%),  **Canada** | Cross-sectional (Survey) | - Income - Hours per week - Hours on call - Consultations per week | - There is a persistent gap in earnings of family physicians in Ontario, even when controlling for family characteristics. - Women work about 80% the number of hours as men. - Women see a lower volume of patients but spend more time on call | [21] |
| **Bensing** (1993) | To examine whether 1) Female GPs see more female patients than their male colleagues in the same practice; 2) Female GPs are confronted with different health problems than their male colleagues; and 3) Female GPs provide different services to their patients. | 21 group practices with 27 male and 23 female PCPs (covering 47,254 consultations) (N/A), **Netherlands** | Cross-sectional (Prospective primary data collection) | - Patient characteristics - Length of consultation - Problems presented and diagnoses - Services provided | - Contacts with female patients comprise more of a female PCPs workload compared to men - Female PCPs are presented with a different set of medical problems than male PCPs - Women order more lab tests, write fewer prescriptions and do fewer technical interventions. They do more counseling. - Female PCPs spend more time with their patients and have a stronger tendency to provide continuity of care. A part-time effect was observed in most issues studied | [34] |
| **Bergeron** (1999) | To identify major factors that influence physicians home care practices in Quebec. | 487 PCPs in Quebec City (70.0%)  **Canada** | Cross-sectional (Survey) | - Home visits per week - Time spent on home care per week | - Men make more home visits that women; however they spend the same amount of time overall on those home visits. | [36] |
| **Boerma** (2000) | To examine whether male and female GPs in Europe differ in their personal and work-related characteristics, and do male and female GPs in Europe differ in their curative and preventive service profiles. | 8,183 PCPs across 32 European countries (approximately 50%, ranging from 30-87% depending on the country),  **Europe** | Cross-sectional (Survey) | - Office contacts per day - Time allocated per patient - Solo vs. group practice - House calls and after-hours services - Patient age and medical need - Practice location Scope of practice | - Men work more hours on average and excluding on call - Women allocate more time per patient - Increased numbers of female GPs will encourage the establishment of group practices. For women, the flexibility of part- time work, the more limited commitment, and the possibility of salaried employment will often be more attractive. - Female GPs were less involved in activities outside regular office hours - Male PCPs have more contacts per day; however, female PCPs who work full time have significantly more contacts than men who work full time | [18] |
| **Brett** (2009) | To ascertain the retirement intentions of a cohort of Australian general practitioners. | 178 PCPs (37%),  **Australia** | Cross-sectional (Survey) | - Intention to work in general practice until retirement - Reasons for retiring before age 65 years | - 63% of GPs intended to work to at least age 65 years, with men more likely to retire early | [37] |
| **Britt** (1996) | To assess differences between male and female GPs in practice style, the reasons patients consult them, or the nature of medical conditions they manage. It also considered the extent to which differences are accounted for by the effect of confounders | 495 PCPs, all encounters for two 1-week periods (100,000 doctor-patient encounters) (not given),  **Australia** | Cross-sectional (Prospective primary data collection) | - Encounters/week - Home visits - Length of consultation - Conditions managed - Patient characteristics | - Significant differences were found in the work patterns and patient mix of male and female GPs even after adjustment for confounders - Female GPs managed more female-specific endocrine, general, and psychosocial problems. Although male PCPs managed more cardiovascular, musculoskeletal, male genital, skin, and respiratory problems at the univariate level, these differences were no longer apparent after adjustment | [31] |
| **Canadian Institute For Health Information** (CIHI) (2001) | This main focus of this report is to examine fee-for-service physician demographic and  practice characteristics and in particular, changes in physician activity levels between  1989/90 and 1998/99. | 27,660 physicians from the National  Physician Database , (NA),  **Canada** | Longitudinal (Administrative) | - Physician activity ratios | - For female PCPs physicians in 1998/99, activity ratio ranged between 71 and 78% of their male counterparts | [30] |
| **Carek** (2003) | To evaluate the practice profiles of all female family physician graduates of South Carolina training programs compared with male graduates. | 714 PCPs (55%),  **United States** | Cross-sectional (Survey) | - Practice arrangement - Practice location, Practice composition - Hours per week, on call - Number of procedures | - The practice arrangements and settings, on-call frequency, and whether they serve in a medically underserved or physician manpower shortage area did not differ significantly between female and male family physicians - The demographics of female and male family physician practices in regards to age groups of the patients (ie, adolescent, adult, and geriatric patients) are comparable - Female graduates spent less of their time in hospital practice and did procedures less frequently | [23] |
| **Chambers** (1996) | To compare male and female general practitioners with respect to their job satisfaction and professional commitments within and outside their practices. | 620 PCPs (69%),  **United Kingdom** | Cross-sectional (Survey) | - Whether personal responsibility was taken for twelve different tasks - Professional commitments outside practice | - Female doctors were more likely to be working in training practices, and were likely to be on-call less and to work fewer sessions - Male general practitioners were more likely to take lead responsibility for practice computers, minor surgery, meeting external visitors and finance, whereas female practitioners were more likely to be responsible for looking after women patients' health | [46] |
| **Chan** (2003) | To examine factors affecting referrals by primary care physicians to specialists. | All fee-for-service physicians in Ontario for 1997/8,  **Canada** | Cross-sectional (Administrative) | - Rate of referrals | - Female and male physicians had similar referral rates in the bivariate analysis, but females had higher referral rates after controlling for patient age and gender | [35] |
| **Chaytors** (2001) | We compared the types of procedures performed and obstetrical care provided by family practice residency graduates, by practice location and physician gender | 442 PCPs (63%),  **Canada** | Cross-sectional (Survey) | - Top five procedures for family practice graduates, according to sex and practice location (rural vs. urban) | - Significantly more male graduates reported performing most procedures (including, except for IUD insertion, which were performed by relatively more females - A significantly greater proportion of female graduates performed or were skilled in most obstetrical care practices, except for forceps deliveries, which did not differ by gender - Few gender differences were noted in the performance of procedures in rural areas | [32] |
| **Cohen** (1991) | To examine the effect of physician gender on billings for a population of GP grads of McMaster University using the Ontario Health Insurance plan billing data. | 322 PCPs, all graduates of McMaster University (N/A),  **Canada** | Cross-sectional (Administrative) | - Months billed per year - Number of patients per month - Number of services per patient - Patient gender Patient age | - Women were more likely to working part time, billed fewer months of the year, and saw fewer patients - Women provided more psychotherapy and counseling and ordered more laboratory tests - Women provided fewer hospital, emergency room, and intrapartum services | [25] |
| **Constant** (2008) | To estimate the impact of an increasing share of female physicians on the total output of Canadian physicians. | All Canadian physicians, 1989-1998 (N/A)  **Canada** | Cross-sectional (Administrative) | - Number of services per year | - Female physicians provide fewer services than male counterparts. The gender gap reaches its peak for physicians aged 36 to 40 years, and then decreases thereafter. | [14] |
| **Cree** (2001) | To evaluate the gender-related impact on services provided by general practitioners in Alberta. | All PCPs practicing in Alberta (N/A),  **Canada** | Longitudinal (Administrative) | - Physician income | - More male than female PCPs appear in high billing categories - Female general practitioners more commonly provided a high proportion of in- office services and saw fewer patients per day. The practices of female general practitioners, relative to those of their male counterparts, contained, on average, a higher proportion of female patients and a lower proportion of senior patients and rural patients - As we approach a 50/50 split in the number of male vs. female general practitioners, we forecast an adequate supply of major assessment and counseling services and a shortfall of other assessment services | [47] |
| **Crossley** (2009) | To examine the relative importance of different factors in explaining changes in the number of hours spent in direct patient care by Canadian general/family practitioners over the period 1982–2003. | 45,362 observations total across all survey years (30-80% depending on the year),  **Canada** | Longitudinal (Survey) | - Hours of direct patient care over time. | - For male PCPs: that there is little age effect on hours of direct patient care, there is no strong cohort effect on hours of direct patient care, but there is a secular decline in hours of direct patient care over the period. - Female physicians on average work fewer hours than male physicians - For female GPs: there is a clear age effect on hours of direct patient care, there is no strong cohort effect, and there has been little secular change in average hours of direct patient care - The changing behaviour of male GPs accounted for a greater proportion of the overall decline in hours of direct patient care than did the growing proportion of female GPs in the physician stock | [28] |
| **Dumontet** (2012) | The objective of this study is to measure and analyse the 2008 income gap between males and females general practitioners (GPs). | 339 PCPs (11.3%)^1^,  **France** | Cross-sectional (Survey) | - Income - Number of consultations per year - Number of hours worked per day - Working days per week - Group or solo practice - Frequency of gynecologic and obstetric visits | - 61% of the gender income gap is explained by the gender differences in workload, i.e., number of consultations and visits, which is on average significantly lower for female GPs than for male GPs. - Male and female PCPs are equally likely to work in group practices - Female GPPs perform gynecologic and obstetric follow-ups more frequently - Female GPs have a higher marginal return in terms of earnings when performing an additional medical services | [2] |
| **French** (2006) | To explore gender differences in contractual commitments and job satisfaction among GP principals. | 942 GP Principals (50%)  **United Kingdom** | Cross-sectional (Survey) | - Part time vs fill time status - Out of hours care provision | - Males were less likely to work part time and more likely to participate in out-of-hours work. This result was unaffected by practice size or location. | [40] |
| **Gravelle** (2007) | To compare the reported hours worked by GPs with that of other professions and to explain the variation in GP hours worked and on call. | 2166 PCPs (52%),  **United Kingdom** | Cross-sectional (Survey) | - Part time vs full time status - Hours worked - Hours on call | - The sex differences between GPs in hours worked are mostly attributable to the differential impact of family circumstances, particularly the number of children they have | [17] |
| **Harrison** (2011) | To examine whether differences between male and female physicians in terms of patient mix, and reasons for encounters found in an earlier study to be inherent differences remain with today’s GPs, or if they have leveled out with the increased numbers of female GPs. | 988 GPs , 98,000 encounters (22.7%)  **Australia** | Cross-sectional (Survey) | - Size of practice (number of GPs) Practice location (rural/urban) - Patient reason for encounter - Problems managed Management method used | - The effect of other GP characteristics, the patient mix and patients’ demand for care account for some of the differences in problems managed by male and female GPs; however, even after controlling for these factors, female GPs remain more likely to manage problems of a psychosocial, female-specific or general nature - Differences in management practices are also inherent to GP sex, with female GPs more often using clinical treatments, pathology tests, referrals and imaging | [1] |
| **Kazanjian** (2000) | To describe the supply and distribution of physicians in BC by specialty; to describe variation in scopes of practice among physicians in BC, to analyze the stability of physician human resources in BC, and to examine age related effects on physician output. | 4335 PCPs (N/A),  **Canada** | Longitudinal (Administrative) | - Herfindal index (measure of the concentration of physicians services) | - Mean HI is lower for women, suggesting that they are less specialized, providing services more consistently across multiple domains. For females, there is no effect on urban rural status. For men, urban physicians have higher Herfindahl index | [48] |
| **Keane** (1991) | This descriptive study examines practice patterns of relatively young female and male GP/FPs trained and practicing in a Canadian setting (Ontario) as fee-for-service practitioners. | 644 PCPs (N/A)  **Canada** | Cross-sectional (Administrative) | - Sex-age structure of practice; - General assessment rates - Out-of-office visits (nonemergency hospital visits; emergency room duty; house calls) - After-hours services - Scope of practice (prenatal care; obstetrical care; chronic care) - Service mix | - There are a number of significant differences between female and male physicians in a sample of relatively young, Ontario-trained PCPs practicing in Ontario concerning their personal characteristics, basic practice descriptors, practice sex-age structure, service mix, and service allocation. - Women provided fewer services, earned less, and saw fewer patients than men. - Fewer women than men billed for obstetrical care and after-hours visits - More women than men billed for psychotherapy, and prenatal care, but prenatal care less hospital work of both types, and fewer house calls and after-hours visits than men | [24] |
| **Leese** (2002) | To explore gender and career-stage differ ences in factors affecting GP retention, and to draw out lessons for enhancing the stock of GPs across the UK and Europe. | 621 PCPs who left practice (57%),  **United Kingdom** | Cross-sectional (Survey) | - Age upon leaving practice - Impact of family characteristics on leaving | - Compared with male leavers, females were more likely to have practiced part-time, in inner city rather than rural areas - Female leavers were also more likely to be younger, married to a doctor and to have children under the age of 18 - There were, however, no gender differences in terms of partnership size, out-of-hours arrangements, or marital status | [38] |
| **Mayorova** (2005) | Do graduated male and female GPs differ in their preferences of work arrangements (practice form, practice size and practice establishment)?  (b) How have these male/female preferences developed between 1982 and 2001? | 7234 PCPs (94%),  **The Netherlands** | Longitudinal (Survey) | - Preference for solo practice among graduated GPs trying to find a practice. Preference for the size of practice - Preference for practice establishment. | - Female PCPs are less likely to prefer a solo practice; however, there is a decrease in preferences for a solo practice and decrease is more substantial for men than for women - Compared to female GPs male doctors more frequently prefer a practice of at least 2500 patients - Men prefer taking over an existing general practice, while women prefer more to associate with already established physicians | [49] |
| **McKinstry** (2006) | To discover how male and female general practice performers (formerly general practice unrestricted principals (henceforth referred to as GP principals) and non-principals) divided their time between general medical service (GMS) activity and other activities such as teaching and administrative tasks. | 2541 for PCP principals (67.2%) and 749 for non principals (65.2%),  **United Kingdom** | Cross-sectional (Survey) | - Number of sessions per week on GMS and non-GMS activities principals non principals by age - Intention to retire | - Women doctors in primary care in Scotland work fewer hours in all age groups than their male counterparts - Auxiliary activities such as teaching and administrative duties are not being taken up by women as often as men | [39] |
| **McMurray** (2002) | To determine the impact of increasing numbers of women in medicine on the physician work force in Australia, Canada, England, and the United States. | Sample size and response rate not presented.  **Australia, Canada, United Kingdom, United States** | Cross-sectional (Survey) | - Hours per week | - In Australia, Canada and the US, male PCPS work on average 50, 55, and 55 hours per week, while female PCPs work 34, 46, and 49 hours per week | [22] |
| **Norton** (1994) | To examine the impact of a variety of demographic factors on primary care practice patterns. | 456 PCPs (33%)^2^,  **Canada** | Cross-sectional (Survey) | - Number of office visits per week - Number of hours spent in office each week - Patient care activities outside the office | - The increasing number of female physicians has possible ramifications: there will be a decrease in services provided in the office - Female physicians engage in fewer activities out- side the office so there will be a significant decrease in the number of physicians providing out-of-office services and, as a result, in the number of services provided. | [19] |
| **Raymont** (2005) | To describe the characteristics and workload of New Zealand general medical practitioners (GPs). | 244 PCPs  (63%),  **New Zealand** | Cross-sectional (Survey) | - Days worked per week - Patients seen per day and per week - After hours work - Time on call | - Relative to males, female practitioners worked fewer days per week, saw fewer patients, per week, and had a lower work rate per day | [26] |
| **Slade** (2002) | To describe physician workload measures in relation to a comprehensive set of demographic variables. | 3004 PCPs (58%),  **Canada** | Cross-sectional (Survey) | - Weekly work hours - Services offered - Clinical procedures performed. | - Male respondents reported working, on average, 8.9 more total hours per week, excluding on call time - No differences were found between male and female physicians in terms of mean number of medical services offered and clinical procedures performed - No difference in the proportion who offer obstetrical care | [20] |
| **Ubokudom** (1997) | What gender-related differences are there among primary care physicians, and what are the implications of the similarities and differences among male and female physicians for health care reform? | 385 PCPs (52%),  **United States** | Cross-sectional (Survey) | - Practice characteristics - Practice environments - Administrative burden | - Female physicians are more likely than male physicians to practice in the more densely populated and lower per capita income counties, but the differences are not statistically significant - Female physicians are more likely to practice in the physician-rich and higher Medicaid-eligible population counties than male physicians, and the differences are statistically significant | [50] |
| **Watson** (2006) | This study quantifies temporal shifts in  workload (number of hours and volume of ser- vices) among general and family practitioners (GP/FPs) in Canada. It also assesses the use of their services. | Survey: 1,632 PCPs (46%) in 1993 and 1196 PCPs (28%);  Administrative: 10,361 in 1992 and 10,541 in 2001 (90% and 85% of GP population respectively)  **Canada** | Longitudinal (Survey & Administrative) | - Hours worked - Workload (billings) | - Between 1992 and 2001, female GPs reduced FPs reduced their workloads (a 6.1 percent relative decline), while those of male GP/FPs remained relatively stable (a 0.1 percent relative decline) - The result is accentuated sex differences in workloads over time, where females held workloads equivalent to 74 percent of those of their male counterparts in 1992 and 68 percent in 2001 - There are differences in direct patient care work hours per week among male and female GP/FPs who had children under age six while sex differences diminished among males and females who had no dependents | [8] |
| **Weeks** (2006) | The objective of this analysis was to explore the  inﬂuence of race and gender on the incomes of  black and white family physicians, after adjusting for work effort, practice characteristics, and provider characteristics that are likely to inﬂuence physician incomes. | 977 PCPs (786 white males, 20 black males, 159 white females, and 12 black females)(Response rate not provided)  United States | Cross-sectional (Survey) | - Annual income - Number of consultations - Practice location - Serves Medicaid population | - During the 1990s, female gender was associated with lower annual incomes among family physicians, substantially so for black women - Black male family physicians reported seeing 2% more visits and working 11% more hours than their white male counterparts, white and black women reported seeing 25% and 39% fewer visits, respectively, and working 12% and 17% fewer annual hours, respectively, than white men | [51] |
| **Weyrauch** (1995) | To identify differences in the type and amount of work performed by male and female HMO physicians. | 21 PCPs at one HMO clinic (100%),  **United States** | Cross-sectional (Survey) | - Patient encounters per day - Self-assessed and encounter-form based workload Ambulatory care group panel evaluation Qualitative observation of practice patterns | - The kind of work male and female physicians did differed on the bases of the encounter-form based diagnoses, ambulatory care group panel assessments and participant observation - The amount of work performed by each gender did not appear to be different when evaluated be age-sex-adjusted panel size, patient encounters per unit time, a subjective magnitude estimation workload questionnaire and an encounter form-based workload assessment | [43] |
| **Woodward** (1995) | To examine differences in activity between male and female physicians after accounting for physician and practice characteristics, and characteristics of the market area in which the practice is located. | 2771 PCPs and GPs (N/A/)  **Canada** | Longitudinal (Administrtive) | - Part time vs full time status - Number of patient encounters and services per year - Annual income (billings) - Practice characteristics | - Female family physicians and GPs provide fewer services, see fewer patients, bill less, and deliver fewer services per patient. - More women are in group practice. - Age-sex interaction was not significant for any measures of activity. | [29] |

^1^The inclusion period was closed after four months, at which point, 423 questionnaires out of 3000 sent had been collected, with 339 usable.

^2^The Peer Assessment Program selected 1384 PCPs for assessment. Of these 59% were excluded or exempted, leaving 547. An additional 91 were excluded for being over age 69.
